# Microvasculature and intraplaque hemorrhage in atherosclerotic carotid lesions: a cardiovascular magnetic resonance imaging study

**DOI:** 10.1186/s12968-019-0524-9

**Published:** 2019-03-04

**Authors:** Geneviève A. J. C. Crombag, Floris H. B. M. Schreuder, Raf H. M. van Hoof, Martine T. B. Truijman, Nicky J. A. Wijnen, Stefan A. Vöö, Patty J. Nelemans, Sylvia Heeneman, Paul J. Nederkoorn, Jan-Willem H. Daemen, Mat J. A. P. Daemen, Werner H. Mess, J. E. Wildberger, Robert J. van Oostenbrugge, M. Eline Kooi

**Affiliations:** 1 Department of Radiology and Nuclear Medicine, Maastricht University Medical Centre, P.O. Box 5800, 6202 AZ Maastricht, The Netherlands; 2 CARIM School for Cardiovascular Diseases, Maastricht University, Maastricht, The Netherlands; 3 Department of Neurology & Donders Institute for Brain Cognition & Behaviour, Radboud University Medical Centre, Nijmegen, The Netherlands; 4 Department of Neurology, Maastricht University Medical Centre, Maastricht, The Netherlands; 5 Department of Epidemiology, Maastricht University, Maastricht, The Netherlands; 6 Department of Pathology, Maastricht University Medical Centre, Maastricht, The Netherlands; 7 Department of Neurology, Academic Medical Centre, Amsterdam, The Netherlands; 8 Department of Surgery, Maastricht University Medical Centre, Maastricht, The Netherlands; 9 Department of Pathology, Academic Medical Centre, Amsterdam, The Netherlands; 10 Clinical Neurophysiology, Maastricht University Medical Centre, Maastricht, The Netherlands

**Keywords:** Atherosclerosis, DCE-MRI, Intraplaque hemorrhage, Microvasculature, Ischemic stroke, Cardiovascular Disease, Magnetic Resonance Imaging (MRI), Cerebrovascular Disease/Stroke, Transient Ischemic Attack (TIA)

## Abstract

**Background:**

The presence of intraplaque haemorrhage (IPH) has been related to plaque rupture, is associated with plaque progression, and predicts cerebrovascular events. However, the mechanisms leading to IPH are not fully understood. The dominant view is that IPH is caused by leakage of erythrocytes from immature microvessels. The aim of the present study was to investigate whether there is an association between atherosclerotic plaque microvasculature and presence of IPH in a relatively large prospective cohort study of patients with symptomatic carotid plaque.

**Methods:**

One hundred and thirty-two symptomatic patients with ≥2 mm carotid plaque underwent cardiovascular magnetic resonance (CMR) of the symptomatic carotid plaque for detection of IPH and dynamic contrast-enhanced (DCE)-CMR for assessment of plaque microvasculature. *K*^trans^, an indicator of microvascular flow, density and leakiness, was estimated using pharmacokinetic modelling in the vessel wall and adventitia. Statistical analysis was performed using an independent samples T-test and binary logistic regression, correcting for clinical risk factors.

**Results:**

A decreased vessel wall *K*^trans^ was found for IPH positive patients (0.051 ± 0.011 min^− 1^ versus 0.058 ± 0.017 min^− 1^, *p* = 0.001). No significant difference in adventitial *K*^trans^ was found in patients with and without IPH (0.057 ± 0.012 min^− 1^ and 0.057 ± 0.018 min^− 1^, respectively). Histological analysis in a subgroup of patients that underwent carotid endarterectomy demonstrated no significant difference in relative microvessel density between plaques without IPH (*n* = 8) and plaques with IPH (*n* = 15) (0.000333 ± 0.0000707 vs. and 0.000289 ± 0.0000439, *p* = 0.585).

**Conclusions:**

A reduced vessel wall *K*^trans^ is found in the presence of IPH. Thus, we did not find a positive association between plaque microvasculature and IPH several weeks after a cerebrovascular event. Not only leaky plaque microvessels, but additional factors may contribute to IPH development.

**Trial registration:**

NCT01208025. Registration date September 23, 2010. Retrospectively registered (first inclusion September 21, 2010).

NCT01709045, date of registration October 17, 2012. Retrospectively registered (first inclusion August 23, 2011).

## Background

Rupture of a vulnerable atherosclerotic plaque is very likely to be an important cause of clinical ischemic events such as stroke or myocardial infarction [1–3]. The presence of intraplaque haemorrhage (IPH) has been identified as a key pathological factor that is associated with plaque rupture, plaque progression, and predicts cerebrovascular events [4–9]. The predictive value of carotid IPH on cardiovascular magnetic resonance (CMR) for cerebrovascular events was confirmed in several meta-analyses [5–7], showing a hazard ratio of 5.7 (95% confidence interval of 3.0 to 10.9) [5]. However, the pathophysiological origin of IPH is poorly understood. The dominant view is that IPH originates from erythrocytes that extravasate from plaque microvessels into the plaque [10, 11]. These microvessels, mainly originating from the adventitia, have poorly formed endothelial cell junctions, making them prone to leakage of erythrocytes and inflammatory cells into the plaque [10]. Neovessel sprouting from the lumen into the plaque tissue has also been identified and could play a contributing role to IPH [12, 13]. Alternatively, it has been suggested by us and others that plaque rupture/fissure is important in the development of IPH [2, 14, 15]. Indeed, recently we found that IPH occurs often in the proximity of fissures in the fibrous cap (FC) [16].

CMR has been established as the preferred imaging method for non-invasive in vivo detection of IPH [17–19]. In addition, dynamic contrast-enhanced (DCE)-CMR has emerged as a non-invasive method to assess carotid plaque microvasculature. Quantitative pharmacokinetic DCE-CMR parameters, in particular *K*^trans^ (a reflection of the microvessel flow, density, and permeability), correlate well with the extent of plaque microvasculature on histology as demonstrated by ourselves and others [20–23]. In addition, it has been shown that *K*^trans^ can be determined with a good inter-scan reproducibility (ICC of 0.79, *p* < 0.05 and coefficient of variation of 16% for the Patlak model) [23]. Since CMR allows non-invasive assessment of IPH and plaque microvasculature, it is uniquely suited to investigate the relation between microvessels and IPH in patients. DCE-CMR has also the advantage that *K*^trans^ is not only dependent on microvessel density, but also on plaque microvasculature flow and leakiness. Moreover, CMR allows to study patients with a mild to moderate stenosis, who rarely undergo carotid endarterectomy so that these plaques are usually not available for histopathological studies. A recent CMR study revealed a positive association between adventitial *K*^trans^ and the presence of IPH within a group of 27 symptomatic patients with carotid plaque and varying degree of stenosis [24]. These findings are in line with the prevailing hypothesis that IPH originates from leaky plaque microvasculature. However, confirmation of these results in a larger patient group is needed.

The aim of the present study was to investigate whether there is an association between the atherosclerotic plaque microvasculature and presence of IPH in a relatively large prospective cohort study in symptomatic patients with carotid plaque.

## Materials and methods

### Study population

In the present study, consecutive symptomatic patients with a recent ischemic stroke/transient ischemic attack (< 3 months) and a carotid plaque with a thickness of at least 2–3 mm were considered for inclusion in a prospective, observational imaging study. Degree of stenosis was determined with clinically obtained Doppler ultrasound on the ipsilateral side.

Patient demographics and clinical risk factors were collected during follow-up. Patients with a probable cardiac source of embolism, clotting disorder, severe comorbidity, and standard contra-indications for CMR, such as ferromagnetic/other electronic implants were excluded. Patients with severe renal disease (estimated glomerular filtration rate < 30 ml/min) were not eligible for contrast-enhanced CMR and therefore excluded. Approval of the local Institutional Ethical Review Board was obtained and written informed consent was obtained for all patients. The study was registered at ClinicalTrials.gov under NCT01208025 and NCT01709045.

### CMR imaging

CMR imaging was performed on a 3 T whole body CMR system (Achieva, Philips Healthcare, Best, The Netherlands) using a dedicated 8-channel carotid coil (Shanghai Chenguan Medical Technologies Co., Shanghai, China). A previously described multi-contrast CMR protocol [25] was used to study plaque composition, which included the following sequences: 3D time-of-flight (TOF), 2D T1weighted (T1w) inversion recovery turbo field echo (IR TFE), T2 weighted (T2w) turbo spin echo (TSE) and pre-and post-contrast T1w quadruple inversion recovery (QIR) TSE. The CMR protocol is listed in Table 1. In short, the acquired and reconstructed resolution was 0.62 mm × 0 .62–0.67 mm and 0.30 mm × 0.24–0.30 mm, respectively. For DCE-CMR, an end diastolic electrocardiographically-gated 3D T1w-TFE CMR pulse sequence was acquired centred at the position of the highest plaque burden with the following parameters: repetition/echo time 11.6/5.7 ms, flip angle 35°, Field of View 130 × 130 mm, acquisition/reconstruction matrix 208 × 206/512 × 512, five adjoining transversal slices with a slice thickness of 2 mm [26]. The temporal resolution was approximately 20 s per time frame (dependent on heart rate). At the beginning of the third time frame, 0.1 mmol/kg of a small molecular contrast agent (GBCA), Gadobutrol (Gadovist, Bayer HealthCare, Berlin, Germany), was injected with a power injector (Spectris Solaris, Medrad, Warrendale, Pennsylvania, USA) at 0.5 ml/sec followed by a 20 ml saline flush at the same rate. DCE-CMR acquisition was continued for six minutes after contrast injection. Positioning of the center of the DCE-CMR slices at the position with the highest plaque burden was done by visual assessment by a trained researcher. Table 1 gives an overview of the used CMR sequences in this study.

**Table 1:** Carotid CMR scan parameters

Pulse sequence	3D TOF	3D T1w IR TFE	T2w TSE	Pre- and post-contrast T1w QIR TSE	3D T1w IR TFE (for DCE-CMR)
Acquisition plane	transversal	transversal	transversal	transversal	transversal
TR (ms)	20	9.1	4800	800	11
TE (ms)	5	5.5	49	10	5.7
TI (ms)	–	304	–	282	–
Flip angle (°)	20	15	–	–	35
Number of slices	15	15	15	15	5
Slice thickness (mm)	2	2	2	2	2
FOV (mm)	160 × 160	160 × 128	160 × 160	160 × 160	130 × 130
Acquired matrix	260 × 258	260 × 204	260 × 252	260 × 240	208 × 206
Reconstruction matrix	528 × 528	528 × 528	528 × 528	528 × 528	512 × 512
ECG triggered	No	No	No	No	Yes, end-diastolic
Fat suppression	Yes	Yes	Yes	Yes	No

*TOF* time of flight, *IR TFE* inversion recovery turbo field echo, *TSE* turbo spin echo, *QIR TSE* quadruple inversion recovery turbo spin echo, *TR* repetition time, *TE* echo time, *TI* inversion time, *FOV* field of view, *ECG* electrocardiogram

### CMR image review

**Fig. 1:**
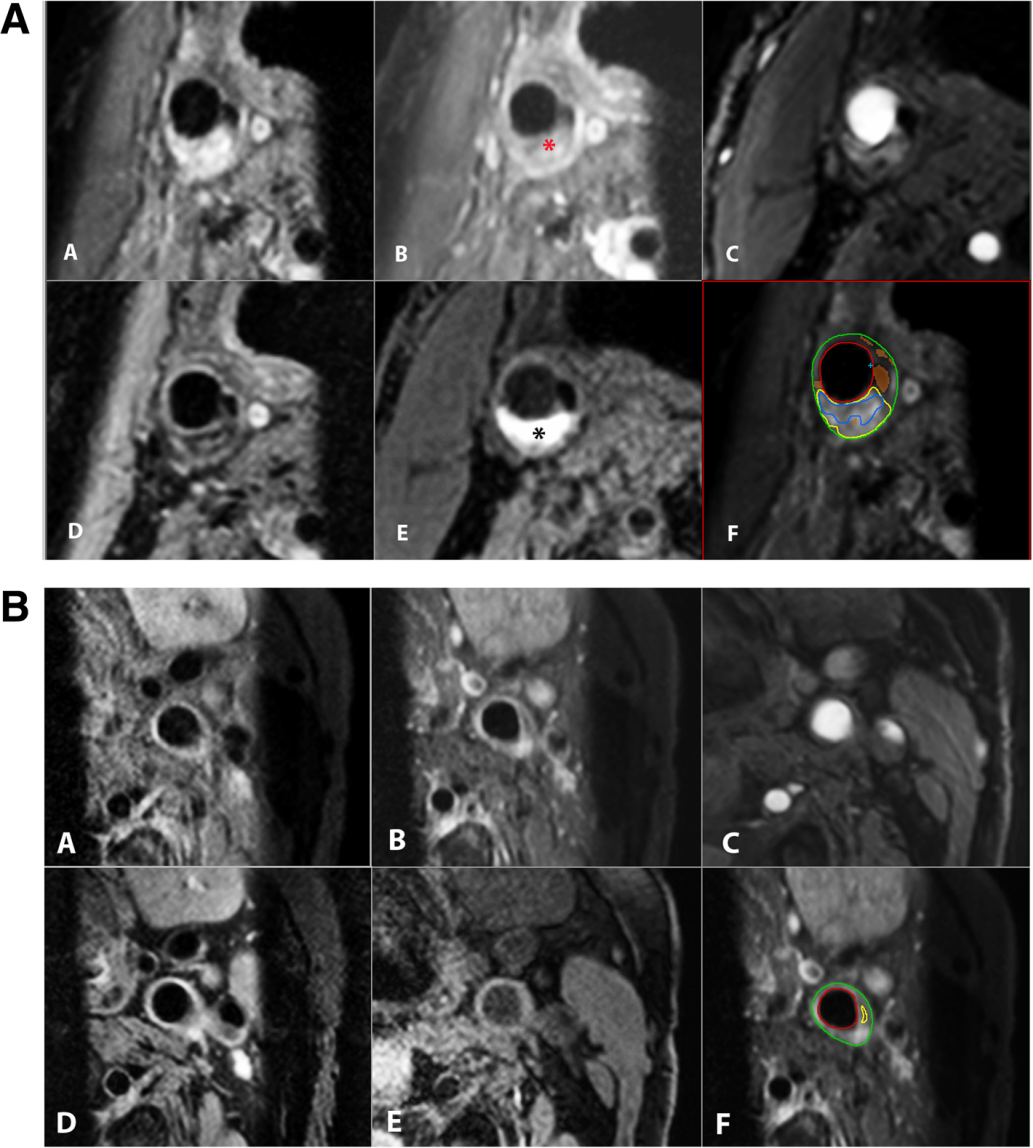
**a** Transversal cardiovascular magnetic resonance (CMR) images of a patient with carotid plaque in the right carotid artery with intraplaque hemorrhage (IPH). The following CMR sequences were acquired: (A) pre-contrast T1-weighted (T1W) quadruple inversion recovery (QIR) turbo spin echo (TSE), (B) post-contrast T1W QIR TSE, (C) time of flight (TOF), (D) T2W TSE and (E) T1W inversion recovery (IR) turbo field echo (TFE). Three regional saturation slabs were positioned on T1W QIR TSE and T2W TSE sequences; one on the throat to reduce swallowing artefacts and another two on the subcutaneous fat, with an angle of approximately 45 degrees with respect to the regional saturation slab that is positioned on the throat (both left and right) to reduce ghosting. A lipid-rich necrotic core was identified as a region within the bulk of the plaque that does not show contrast enhancement (* on B) on the post-contrast T1W QIR images. On the T1W IR TFE image, a hyper-intense signal in the bulk of the plaque can be clearly observed, indicating the presence of IPH (* on panel E). Panel F shows the plaque contours on the post-contrast T1W QIR TSE images (green = outer vessel wall, red = inner vessel wall, yellow = lipid-rich necrotic core, blue = IPH, orange/brown = calcifications). **b** Transversal CMR image of a carotid plaque in the right carotid artery without IPH but with a small lipid-rich necrotic core. All panels consist of the same sequences as in Fig. 1a

Contours of the plaque and plaque components were drawn on the ipsilateral side by a single experienced observer as described previously, using dedicated vessel wall analysis software (VesselMass, Leiden, the Netherlands) [26]. In case of doubt, a second highly experienced observer was consulted. Both readers were blinded for clinical characteristics and DCE-CMR test results. Per slice, luminal and outer vessel wall contours are annotated on the pre-contrast QIR T1w TSE images. In case of artefacts in the pre-contrast images, the post-contrast QIR T1w TSE, T1w TFE or T2w TSE are used, subsequently. Normalized wall index (NWI), an indicator of plaque burden, was calculated as total vessel wall area divided by the sum of the luminal and vessel wall area on the T1w QIR sequence. IPH was scored present by trained readers if a hyperintense signal (compared with the adjacent sternocleidomastoid muscle) was observed on the T1w TFE or TOF images in the bulk of the plaque, as previously described [25, 27, 28]. Fig. 1 illustrates a plaque with IPH (Fig. 1a) as well as a plaque without IPH (Fig. 1b). The lipid-rich necrotic core is also delineated on the CMR images (by comparison of pre- and post-contrast T1W QIR images; in case of absence of post-contrast images, the T2w TSE images were used), using previously published criteria [29–31]. On the post-contrast T1w TSE image, the FC can be identified as a high signal area between the lipid-rich necrotic core and the lumen of the carotid artery [2]. The FC was dichotomized according to previously published categorization [12]. When a continuous high signal area between IPH and the lumen was identified, FC status was classified as being ‘thick and intact’. When no or an interrupted high signal area was identified, FC status was classified as begin ‘thin and/or ruptured’.

### DCE-CMR image review

Luminal and outer vessel wall contours were transferred to the DCE-CMR images and for each time frame adjustment of these contours was deemed necessary when small patient displacements had occurred during the dynamic acquisition. To avoid partial volume effects, luminal contours were corrected by keeping sufficient distance from the vessel lumen, as described in previous studies [32]. When the adventitial vasa vasorum showed hyperenhancement after contrast material administration, outer plaque contours were corrected to include the adventitial vasa vasorum. The entire vessel wall region is defined as the region between the luminal and outer wall contours. The adventitial region of the vessel wall was delineated according to previously described criteria, i.e. all pixels within 0.625 mm of the outer wall contour in a region of the vessel wall with plaque (defined as having a wall thickness larger than 1.5 mm) [24].

### Carotid endarterectomy and histological preparation

**Fig. 2:**
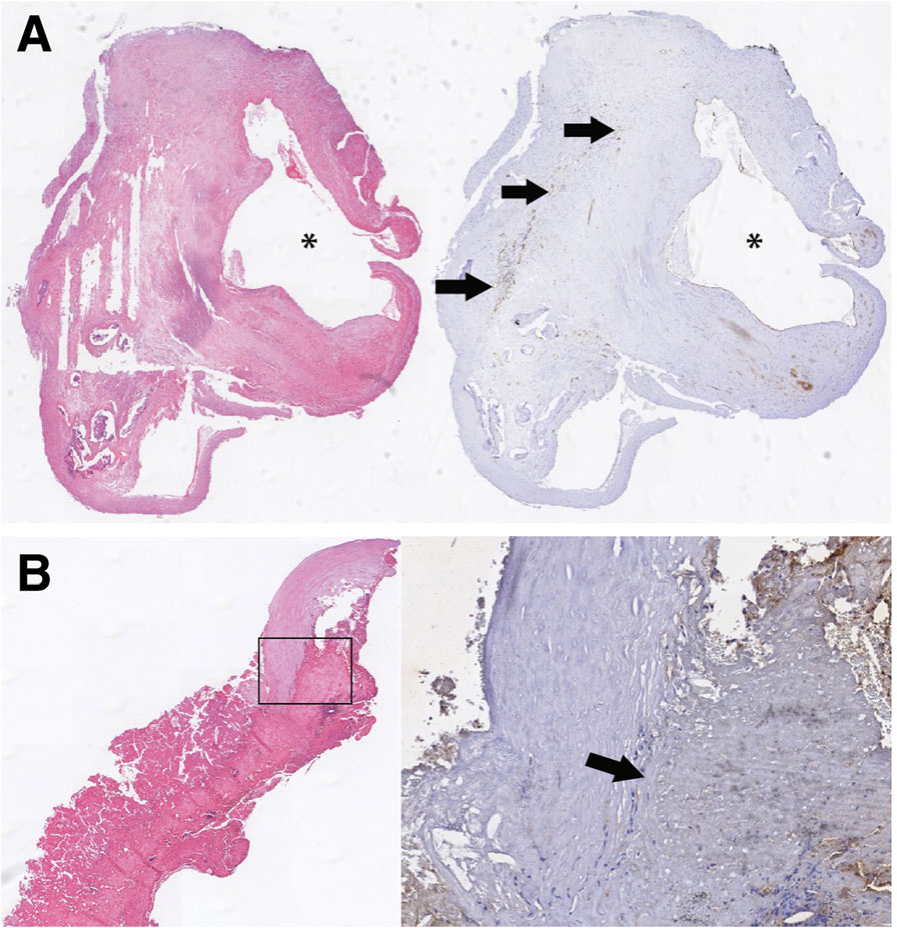
**a** Hematoxylin and eosin (HE) staining (demonstrating the absence of IPH) and the corresponding CD 31 staining (black arrows show presence of microvessels) from a histological specimen obtained during carotid endarterectomy. **b** Hematoxylin and eosin (HE) staining (demonstrating IPH) and the corresponding CD 31 staining (no presence of microvessels) from a histological specimen obtained during carotid endarterectomy, the black arrow points towards the area were intraplaque hemorrhage is present

Indication for carotid endarterectomy (CEA) was based on the clinician’s decision. Surgeons were instructed to remove the carotid plaque in one piece. Carotid endarterectomy specimens were collected after surgery and histological processing was performed as described previously [23]. In short, after CEA the carotid plaques were immediately fixed in 10% buffered formalin, transversely cut in 3-mm slices, decalcified, embedded in paraffin, and cut in 4-mm (transverse) slices. Plaque microvasculature was detected with immunohistochemistry using primary antibodies against CD31 (clone JC70A, Dako North America, Carpinteria, California, USA) for identification of endothelial cells. Plaque microvasculature was quantified on high spatial resolution digital images by using morphometric analysis software (QWin V3, Leica, Cambridge, United Kingdom). Plaque microvasculature was measured as the CD31-positive area surrounding a lumen. Relative density of microvessel endothelium was calculated by dividing the total CD31-positive area by the total plaque area. Fig. 2 illustrates a representative hematoxylin and eosin (HE) staining (demonstrating IPH) and the corresponding CD 31 staining (demonstrating microvessels in brown) from a patient without (Fig. 2a) and a patient with IPH (Fig. 2b).FC integrity was defined as a disruption of the endothelial layer and evidence of luminal thrombus formation at that location, and was scored by 2 independent readers (vascular biologists) with more than 15 years of experience in carotid plaque scoring. The outcome of FC integrity (intact or disrupted) was reported on the plaque level.

## Data analysis

### Pharmacokinetic modelling

Pharmacokinetic parameters were estimated using the Patlak model [33] on a voxel-wise basis as previously described [23] with a phase-based population averaged vascular input function (VIF) determined in the carotid artery [26]. Shortly, GBCA concentrations in the plaque were calculated from the signal intensity time course by using the Ernst equation based on literature values for the longitudinal and transversal relaxation times of tissue [34] and the r_1_ and r_2_ relaxation rates of contrast medium (CM) [35] similar as in previous studies [23, 24, 36, 37].

### Statistical analysis

For descriptive purposes, nominal and categorical variables are presented as absolute numbers and percentages and continuous variables as mean with standard deviation (if normally distributed) or as median with range (if not normally distributed). Differences in proportions between IPH positive and IPH negative patients were tested for significance using the Chi square test. For testing differences in continuous variables, such as vessel wall *K*^trans^ and adventitial *K*^trans^, the t-test for independent samples was used.

Multivariable linear regression analyses with vessel wall *K*^trans^ or adventitial *K*^trans^ as dependent variable were performed to adjust for differences in baseline characteristics between IPH positive and IPH negative patients. IPH (yes vs. no) and clinical risk factors with a statistically significant association with IPH were entered as independent variables. The regression coefficient corresponding with IPH group represents the adjusted mean difference *K*^trans^ values between IPH positive and IPH negative patients.

*P*-values < 0.05 were considered to indicate statistical significance. SPSS (version 23, International Business Machines, Armonk, New York, USA) was used for the statistical analyses.

## Results

### Patient characteristics

Within the present study, a total of 181 patients were enrolled. Fifteen patients did not receive an CMR scan (claustrophobia: *n* = 5, patient declined CMR: *n* = 3, obesity: *n* = 2, ferromagnetic or other electronic implants: *n* = 2, technical CMR problems: *n* = 1, patient retrieved informed consent: *n* = 1, exclusion because this patient had no carotid plaque *n* = 1). In twenty five patients an DCE-CMR could not be performed due to arrhythmias. Prior to analysis, nine additional patients were excluded due to insufficient CMR image quality. Hence, we could analyse the CMR data of 132 patients (92 male, age: 69.5 ± 8.6 years). Clinical characteristics of these patients are presented in Table 2. The CMR examinations were performed at a median of 28 days with a range of 2–215 days after the ischemic event. IPH was detected in 54 (41%) patients. Histological specimens were collected from 23 patients who underwent CEA. Relative microvessel density could be calculated for all 23 patients, however FC integrity assessment was only possible in 16/23 patients due to fragmentation of the histological slides due to calcifications.

**Table 2:** Patient characteristics

Subjects [n] (%)	Total *N* = 132 (100)	IPH present *N* = 54 (40.6)	IPH absent *N* = 79 (59.4)	p-value
Age [y]	69.5 ± 8.6	69.4 ± 8.8	69.6 ± 8.5	0.918
Male sex [n] (%)	69.2%	45 (83.3)	47 (59.5)	0.004
Body mass index [kg/m^2^]	26.7 ± 3.8	26.3 ± 3.0	27.0 ± 4.3	0.296
Currently smoking^*^ [n] (%)	21.1%	8 (14.8)	20 (25.6)	0.015
Diabetes mellitus [n] (%)	18.0%	10 (18.5)	14 (17.7)	1.000
Hypertension [n] (%)	65.4%	43 (79.6)	44 (55.7)	0.005
Hypercholesterolemia^a^ [n] (%)	51.9%	32 (60.4)	37 (47.4)	0.158
Normalized wall index	0.32 ± 0.23	0.43 ± 0.18	0.24 ± 0.23	< 0.001
Statin use before most recent cerebrovascular event^*^ [n] (%)	58.6%	35 (66)	43 (54.4)	0.209
Time between event and MRI [days] (range)	28 (2–215)	22.5 (2–215)	34 (3–122)	0.676
Degree of stenosis				
mild (0–29%)	0.8%	0	1 (1.3)	0.056
moderate (30–69%)	66.2%	30 (55.6)	58 (73.4)	
severe (> 70%)	31.6%	22 (40.7)	20 (25.3)	
occlusion	1.5%	2 (3.7)	0	
Vessel wall K trans (/min)	0.055 ± 0.015	0.051 ± 0.011	0.058 ± 0.017	0.001
Adventitia K trans (/min)	0.057 ± 0.018	0.057 ± 0.017	0.057 ± 0.018	0.980

^*^Data known for 131 out of 132 patients. ^a^ Data known for 130 out of 132 patients

Data are presented as mean ± standard deviation or n (%)

### The association between IPH and plaque microvasculature

**Fig. 3:**
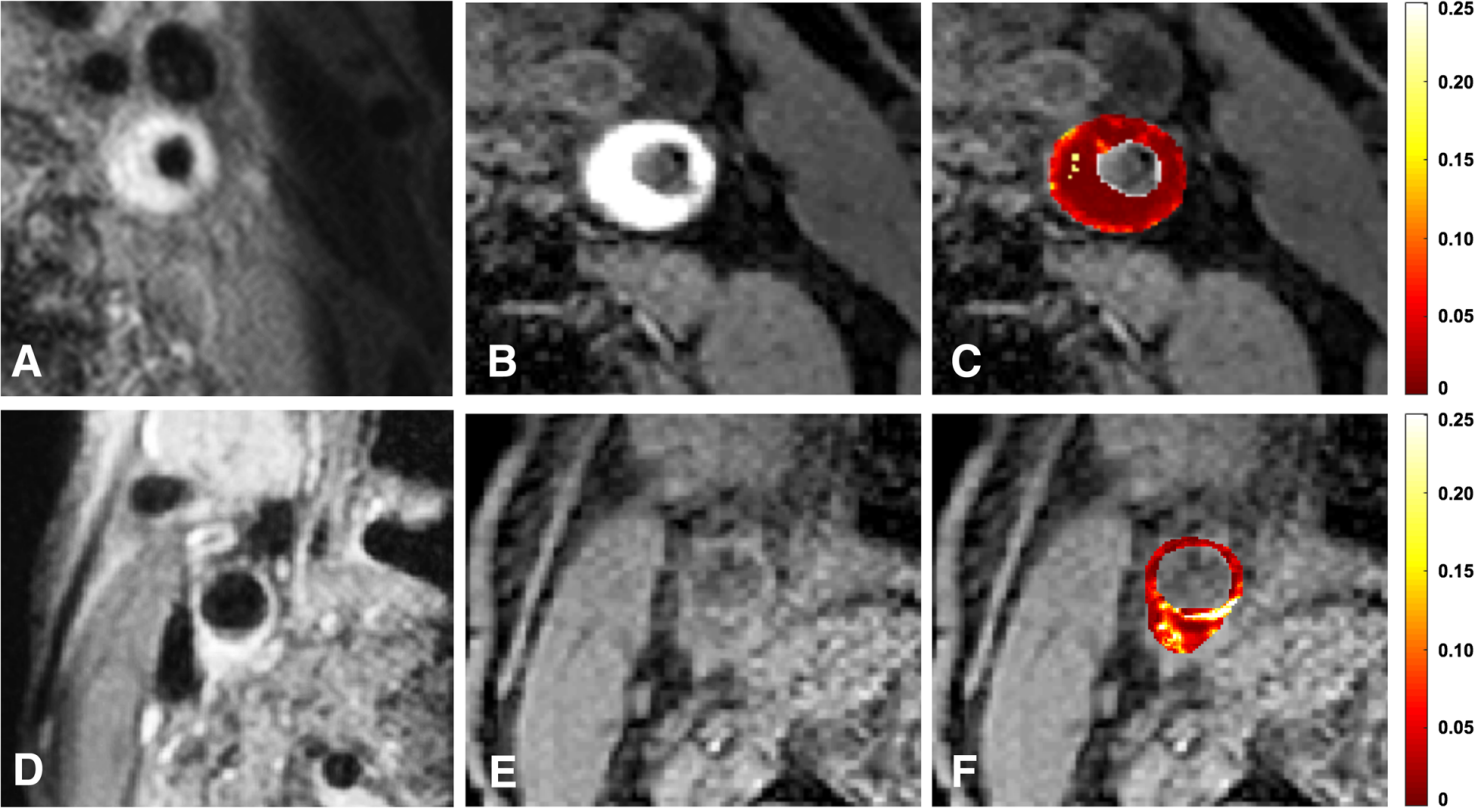
Pre-contrast T1 weighted (T1w) quadruple inversion recovery (QIR) turbo spin echo (TSE) image (**a**) from a patient with intraplaque hemorrhage (IPH). Note that a Regional Saturation Technique (REST) slab is visible on the right side, which was placed over the subcutaneous fat tissue to prevent ghosting artefacts. Three-dimensional T1w inversion recovery turbo field echo (IR TFE) image (**b**) from the same patient with IPH. A hyperintense signal is visible within the bulk the plaque compared with the adjacent sternocleidomastoid muscle (*), indicating the presence of IPH. Parametric *K*^trans^ map of the plaque is overlaid on IR TFE image shown in B (**c**). In this parametric map voxelwise determined *K*^trans^ values are colour encoded from 0 to 0.25 min^− 1^. Within this plaque, the IPH exhibits low *K*^trans^ values, shown in dark red, while higher *K*^trans^ values (brighter red) are observed in the outer vessel wall (adventitial layer). Pre-contrast T1w QIR TSE image from a patient without IPH (**d**). 3D T1w IR TFE image (**e**) from the same patient without IPH. Parametric *K*^trans^ map is overlaid on IR TFE image shown in B (**f**). In this parametric map voxelwise determined *K*^trans^ values are colour encoded from 0 to 0.25 min^− 1^. Within this plaque, higher *K*^trans^ values are observed, shown in bright red/yellow/white. Written informed consent for publication of their clinical details and/or clinical images was obtained from the patients. A copy of the consent forms is available for review by the Editor of this journal

Representative DCE-CMR images are presented in Fig. 3.

**Fig. 4:**
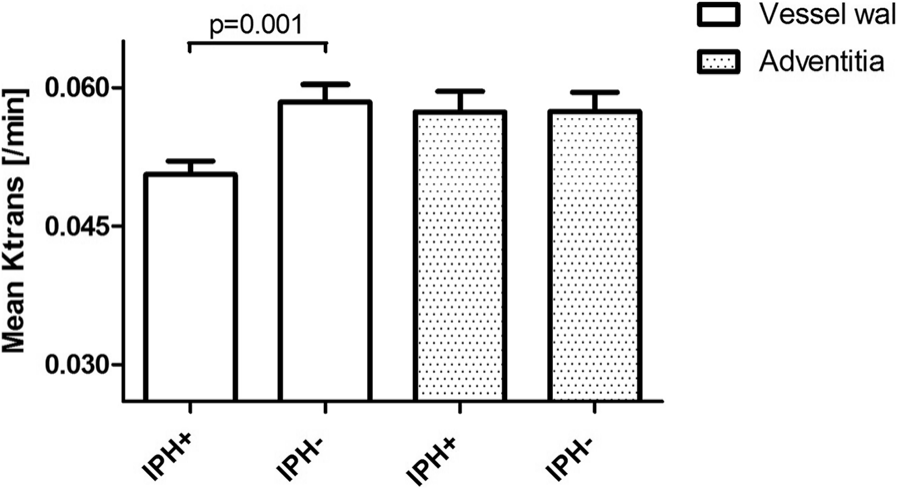
Vessel wall and adventitial *K*^trans^ versus IPH status. Vessel wall and adventitial *K*^trans^ (mean ± standard error) for patients with (IPH+) and without intraplaque haemorrhage (IPH-)

Decreased mean *K*^trans^ of the entire vessel wall was found for patients with IPH (0.051 ± 0.011 min^− 1^) compared to patients without IPH (0.058 ± 0.017 min^− 1^, *p* = 0.001) (Fig. 4, Table 2). No difference was found for mean adventitial *K*^trans^ in plaques with and without IPH (0.057 ± 0.017 min^− 1^ and 0.057 ± 0.018 min^− 1^, respectively). Table 2 shows the differences in relevant baseline characteristics between IPH positive and IPH negative patients. Males, current smokers, hypertensive patients, and a higher NWI showed an positive association with IPH. Adjustment for differences in sex, current smoking, hypertension and NWI using multivariable linear regression analysis resulted in an adjusted mean difference for vessel wall mean *K*^trans^ of − 0.008 (95% CI: -0.13-0.002, *p* = 0.007) and an adjusted mean difference for adventitial mean *K*^trans^ of 0 (95% CI: -0.007-0.007, *p* = 0.925), (Table 3).

**Table 3:** Results of the univariate and multivariate regression analysis

	Univariate			Multivariate		
	Regression coefficient for IPH	95% CI	p-value	Regression coefficient for IPH	95% CI	p-value
Mean vessel wall *K*^trans^	-0.007	−0.13 - -0.002	0.005	−0.008	−0.013 - 0.002	0.007
Mean adventitial *K*^trans^	0	−0.006 - 0.007	0.919	0	−0.007 - 0.007	0.925

Out of the 132 included patients, 59 had a thin or ruptured FC on CMR (44.7%). In the plaques with IPH, in 47 (90.4%) cases a thin or ruptured FC was identified, while in the plaques without IPH only 12 (15.2%) plaques had a thin or ruptured FC (p = 0.001).Histological analysis demonstrated a relative microvessel density of 0.000333 ± 0.0000707 in the group of patients without IPH (*n* = 8), and 0.000289 ± 0.0000439 (*n* = 15) in the IPH positive group. This difference was not significant (*p* = 0.585). Patients who had an intact endothelial layer overlying the FC on histology (*n* = 9) showed higher mean *K*^*trans*^ values (vessel wall *K*^trans^: 0.054/min ± 0.003 versus 0.048/min ± 0.004; *p* = 0.243) adventitial *K*^trans^ (0.063/min ± 0.003 versus 0.050/min ± 0.003, *p* = 0.018) compared to patients with a disrupted endothelial layer overlying the FC. There was no significant difference in microvessel density between patients with or without an intact FC on histological specimens (0.00025 ± 0.00005 versus 0.00030 ± 0.00005, *p* = 0.522) (*n* = 16). There was no significant association between presence of IPH on CMR images and FC integrity assessed on the histopathological specimens (Spearman 0.2, *p* = 0.463, *n* = 16).

## Discussion

The present prospective imaging study in symptomatic patients demonstrated a negative association between vessel wall *K*^trans^, a measure of microvascular density, flow and/or permeability, and the presence of IPH in carotid atherosclerotic plaques. There was no association between adventitial *K*^trans^ and the presence of IPH. We could not confirm a positive association between (leaky) adventitial microvasculature (*K*^trans^) and IPH.

Previously, several histopathological studies reported a link between IPH and increased microvessel density [38, 39]. However, this link was not present in all vascular beds and it was also demonstrated that plaques with IPH with a low microvascular density are quite common. Derksen et al. [38] reported a significantly higher percentage of plaques with increased microvessel density in the presence of IPH (50% versus 42%) in 752 carotid endarterectomy specimens, however no difference was found in 209 femoral endarterectomy plaque specimens. Their study also demonstrated that 50% of the carotid plaques with IPH had a low microvascular density, showing that IPH also occurs frequently in plaques with low microvascular density. McCarthy et al. [39] observed significantly more microvessels in plaques with IPH in a study of 28 carotid endarterectomy specimens, but did not report quantitative data on the increase in plaque microvasculature. Gössl et al. [40] studied microvasculature in 15 hearts, obtained from autopsy and found a positive (Kendall-Tau beta rank) correlation between microvessel density in the coronary vessel wall and glycophorin A score (histological staining for erythrocyte fragments) of 0.65 (*p* < 0.01). It should be noted that also normal vessel wall segments with typically a low glycophorin A score and a low microvasculature density were included in their data analysis, which may have affected the correlation. A recent histopathological study described a non-lipid driven process in which alternative (M2) macrophages promote plaque progression. They demonstrated significantly increased microvessels per unit in areas containing high CD163, a marker of M(Hb) macrophages, suggesting a relation with plaque angiogenesis [41]. Another reason why our results differ from histopathological studies might lay in the difference of how microvasculature was measured. The DCE-CMR parameter *K*^trans^ does not only reflect the microvascular density, but also microvascular flow and leakiness, while in histopathological studies the microvascular density is measured.

Scoring of IPH presence on CMR images has been proven to be an accurate method to identify IPH presence and has been validated with histopathology [17–19, 27]. Reproducibility of the pharmacokinetic variables has been validated in animals, using different contrast media and in patients, showing correlation between *K*^trans^ and microvessel density on histological slices [23, 42–44]. Sun et al. found an increased adventitial *K*^trans^ when IPH was present in a study of 27 symptomatic patients with carotid plaque, while we found no difference in adventitial *K*^trans^ between plaques with and without IPH in our study of 132 patients [24]. Our analysis differed from this previous study because Sun et al. studied the maximum value of mean *K*^trans^ across all slices, which represents microvascular hotspots in the adventitia. A sub-analysis of the 75th percentile value of the *K*^trans^ distribution in the adventitial region in the present study, which is also indicative for hotspots of leaky microvessels, confirmed the lack of a significant difference in adventitial *K*^trans^ plaques with and without IPH. We also looked at *K*^trans^ of the entire vessel, since IPH is located in the bulk of the plaque. We found lower mean *K*^trans^ values in the entire vessel wall in patients with IPH. The lower values of *K*^trans^ in the entire vessel wall may be due to an increased amount of necrotic tissue and thus a decreased microvessel density in plaques with IPH. Indeed, a significant negative Pearson’s correlation coefficient (ρ = − 0.20, *p* = 0.021) was found for mean vessel wall *K*^trans^ with the lipid-rich necrotic core (LRNC) volume. These results indicate a decrease of the atherosclerotic plaque microvasculature in plaques with a larger LRNC. In the aforementioned study of Sun et al., the time interval between carotid CMR and the most recent event was 16.3 ± 18.7 days whereas in the present study this time interval was 34.8 ± 23.3 days. A sub analysis within our study of patients in the group with a time interval below the median (≤28 days, mean 17.4 ± 7.4 days) showed also no significant difference in adventitial *K*^trans^ between IPH positive and IPH negative plaques.

The prevailing hypothesis is that IPH originates from leakage of erythrocytes out of immature microvessels into the atherosclerotic plaque tissue [10]. The present study could not confirm increased microvasculature in plaques with IPH of recently symptomatic patients. In fact, we observed the opposite effect, possibly related to a larger amount of necrotic tissue in plaques with IPH. Additional processes may thus contribute to IPH. It has already been suggested that disruptions of the fibrous cap (e.g. plaque fissures or plaque rupture) can lead to IPH [45]. A histopathological study [14] showed that FC fissures were frequently accompanied by IPH. In line with the concept that IPH can also originate from FC disruption, it was recently shown that the presence of intraplaque haemorrhage is associated with a disruption of the atherosclerotic plaque surface (plaque ulceration and/or a fissured FC) in patients with a mild to moderate carotid stenosis [15]. The CMR results of the present study also demonstrated a higher prevalence of a thin or ruptured FC in patients with IPH. Together these findings support that disruptions of the FC may contribute to IPH [15]. The lack of association that was found in the present study between IPH and FC integrity and microvascular density as assessed on the histopathological specimens may be due to the limited sample size, since only a limited number of patients was scheduled for carotid endarterectomy. Furthermore, the technique used in our study (DCE-CMR) not only looks at microvessel density, like histopathological analysis, but also takes into account flow and leakiness of the microvessels.

Another important factor in the formation of IPH might be plaque biomechanics. This is supported by several findings in literature. Firstly, a recent study in 80 asymptomatic subjects with a 16–79% carotid stenosis showed low diastolic blood pressure to be associated with IPH [46]. Additionally, pulse pressure, which may be considered as driving force for plaque deformation during the cardiac cycle, has been identified as a strong determinant of IPH in 80 human carotid specimens from both symptomatic and asymptomatic patients [47]. Secondly, in histopathological studies IPH occurs more frequently in the upstream region of the plaque, where the blood pressure is much higher, due to pressure wave reflection [48]. Thirdly, computational models have shown IPH to be associated with higher structural wall stress [49]. Finally, a recent study from Lin et al. showed an association between IPH and plaque surface calcification, [50] that has been shown to lead to a local increase in biomechanical stress [51].

Apart from a lower vessel wall *K*^trans^, presence of IPH was positively associated with NWI (indicative for plaque burden). This is in line with previous work, that demonstrated IPH to be associated with wall thickness, plaque length [46], and plaque progression [9, 52–54].

Due to the relatively small size of carotid plaques, a high spatial resolution of DCE-CMR is crucial, limiting the temporal resolution. Accurate determination of the first pass peak of the VIF is not possible with the high spatial resolution CMR pulse sequence. Therefore, within the current study a previously determined population averaged VIF was used. Several studies showed that the use of this population averaged, phase-based VIF is accurate and correlates with microvessel density on histological slices [23, 26, 55, 56].

Scoring of IPH presence on T1-weighted IR TFE CMR images, also known as MP-RAGE images, has proven to be an accurate method to identify IPH presence and has been validated with histopathology by us and others (specificity > 86%, sensitivity ≥80%) [17, 57]. The degradation of haemorrhage into methemoglobin results in T1 shortening and correspondingly causes a high signal intensity on T1-weighted CMR images [58]. Methemoglobin is particular present in fresh and recent intraplaque haemorrhage. Therefore, we cannot exclude that lesions have been classified as IPH negative despite having old IPH. However, previous work from our group has shown that in a similar patient population (stroke patients with ipsilateral < 70% carotid stenosis) the hyperintense signal on IR TFE images is very persistent in time [59]. Out of 92 included patients, a hyperintense signal in the bulk of the plaque on T1w IR TFE CMR images was detected in 20 patients at baseline. One year later, the hyperintense signal was still present in 16 of these 20 patients.Development of the plaque microvasculature is a dynamic process, which is thought to be related to inflammation in the plaque tissue [36, 60, 61]. Increased activity of macrophages within the plaque tissue is associated with hypoxia and subsequent angiogenic stimuli [62, 63] can result in the formation of new microvessels. Previous research has shown that the presence of IPH does not change significantly over a period of up to 1.5 years [53, 54, 64]. This suggest that IPH is either a continuous process or haemoglobin, which produces the hyperintense signal on IR TFE CMR, is entrapped within the atherosclerotic plaque for a long time period. The findings of the present study indicate that there is no continuous leakage of erythrocytes from plaque microvasculature several weeks after an ischemic event, since we found no increase in *K*^trans^ in plaques with IPH at this time point. It cannot be excluded that IPH and the resulting healing process after the bleeding can lead to a reduction in plaque microvasculature.

## Conclusions

In this DCE-CMR study of symptomatic carotid atherosclerosis, we found an inverse association between IPH and vessel wall *K*^trans^ (indicative for microvascular flow, density, and leakiness). This indicates that no on-going leakage of erythrocytes from plaque microvasculature occurs in plaques with IPH several weeks after a cerebrovascular event. This is not in line with the dominant view that IPH develops as a consequence of leaky microvasculature. Additional factors, such as a disrupted plaque surface, may contribute to the development of IPH. Further longitudinal studies are warranted to unravel underlying mechanisms contributing to IPH.

## Abbreviations

AIM-HIGH: Atherothrombosis Intervention in Metabolic Syndrome With Low HDL/High Triglycerides: Impact on Global Health Outcomes
CEA: Carotid enarterectomy
CM: Contrast medium
CMR: Cardiovascular magnetic resonance
CT: Computed tomography
DCE: Dynamic contrast enhanced
ECG: Electrocardiogram
ECST: European Carotid Surgery Trial
FC: Fibrous cap
GBCA: Gadolinium-based contrast agent
ICC: Inter-scan reproducibility
IPH: Intraplaque hemorrhage
IR: Inversion recovery
NASCET: North American Symptomatic Carotid Endarterectomy Trial
NWI: Normalized wall index
QIR: Quadruple inversion recovery
T1w: T1 weighted
T2w: T2 weighted
TFE: Turbo field echo
TOF: Time of flight
TSE: Turbo spin echo
VIF: Vascular input function

## References

[1] Bentzon JF (2014). Mechanisms of plaque formation and rupture. Circ Res.

[2] Falk E (2013). Update on acute coronary syndromes: the pathologists' view. Eur Heart J.

[3] Virmani R (2000). Lessons From Sudden Coronary Death : A Comprehensive Morphological Classification Scheme for Atherosclerotic Lesions. Arteriosclerosis, Thrombosis, and Vascular Biology.

[4] Michel JB (2011). Intraplaque haemorrhages as the trigger of plaque vulnerability. Eur Heart J.

[5] Saam T (2013). Meta-analysis and systematic review of the predictive value of carotid plaque hemorrhage on cerebrovascular events by magnetic resonance imaging. J Am Coll Cardiol.

[6] Hosseini AA (2013). Carotid plaque hemorrhage on magnetic resonance imaging strongly predicts recurrent ischemia and stroke. Ann Neurol.

[7] Gupta A (2013). Carotid plaque MRI and stroke risk: a systematic review and meta-analysis. Stroke.

[8] Kwee RM (2013). MRI of carotid atherosclerosis to identify TIA and stroke patients who are at risk of a recurrence. J Magn Reson Imaging.

[9] Sun J (2012). Sustained acceleration in carotid atherosclerotic plaque progression with intraplaque hemorrhage: a long-term time course study. JACC Cardiovasc Imaging.

[10] Virmani R (2005). Atherosclerotic plaque progression and vulnerability to rupture: angiogenesis as a source of intraplaque hemorrhage. Arterioscler Thromb Vasc Biol.

[11] Moreno PR (2006). Neovascularization in human atherosclerosis. Curr Mol Med.

[12] Horie N (2015). Communication of inwardly projecting neovessels with the lumen contributes to symptomatic intraplaque hemorrhage in carotid artery stenosis. J Neurosurg.

[13] Kumamoto M, Nakashima Y, Sueishi K (1995). Intimal neovascularization in human coronary atherosclerosis: its origin and pathophysiological significance. Hum Pathol.

[14] Constantinides P (1966). Plaque fissures in human coronary thrombosis. Journal of Atherosclerosis Research.

[15] van Dijk AC, et al. Intraplaque hemorrhage and the plaque surface in carotid atherosclerosis: the plaque at RISK study (PARISK). AJNR Am J Neuroradiol. 2015. 10.3174/ajnr.A4414 PMC7964856 26251429

[16] Daemen MJ (2016). Carotid plaque fissure: an underestimated source of intraplaque hemorrhage. Atherosclerosis.

[17] Cappendijk VC (2004). In vivo detection of hemorrhage in human atherosclerotic plaques with magnetic resonance imaging. J Magn Reson Imaging.

[18] Cai JM (2002). Classification of human carotid atherosclerotic lesions with in vivo multicontrast magnetic resonance imaging. Circulation.

[19] Yuan C (2001). In vivo accuracy of multispectral magnetic resonance imaging for identifying lipid-rich necrotic cores and Intraplaque hemorrhage in advanced human carotid plaques. Circulation.

[20] Kerwin WS (2008). MR imaging of adventitial vasa vasorum in carotid atherosclerosis. Magn Reson Med.

[21] Kerwin WS (2006). Inflammation in carotid atherosclerotic plaque: a dynamic contrast-enhanced MR imaging Study1. Radiology.

[22] Kerwin W (2003). Quantitative magnetic resonance imaging analysis of neovasculature volume in carotid atherosclerotic plaque. Circulation.

[23] Gaens ME (2013). Dynamic contrast-enhanced MR imaging of carotid atherosclerotic plaque: model selection, reproducibility, and validation. Radiology.

[24] Sun J (2013). Adventitial perfusion and intraplaque hemorrhage: a dynamic contrast-enhanced MRI study in the carotid artery. Stroke.

[25] Truijman MT (2014). Plaque at RISK (PARISK): prospective multicenter study to improve diagnosis of high-risk carotid plaques. Int J Stroke.

[26] van Hoof RH (2015). Phase-based vascular input function: improved quantitative DCE-MRI of atherosclerotic plaques. Med Phys.

[27] Bitar R (2008). In vivo 3D high-spatial-resolution MR imaging of intraplaque hemorrhage. Radiology.

[28] van den Bouwhuijsen QJ, et al. Change in carotid Intraplaque hemorrhage in community-dwelling subjects: a follow-up study using serial MR imaging. Radiology. 2016:151806. 10.1148/radiol.2016151806 27541684

[29] Cappendijk VC (2008). Comparison of single-sequence T1w TFE MRI with multisequence MRI for the quantification of lipid-rich necrotic core in atherosclerotic plaque. J Magn Reson Imaging.

[30] Cai J (2005). In vivo quantitative measurement of intact fibrous cap and lipid-rich necrotic core size in atherosclerotic carotid plaque: comparison of high-resolution, contrast-enhanced magnetic resonance imaging and histology. Circulation.

[31] Kwee RM (2009). Reproducibility of fibrous cap status assessment of carotid artery plaques by contrast-enhanced MRI. Stroke.

[32] Chen H (2014). Scan-rescan reproducibility of quantitative assessment of inflammatory carotid atherosclerotic plaque using dynamic contrast-enhanced 3T CMR in a multi-center study. J Cardiovasc Magn Reson.

[33] Patlak CS, Blasberg RG, Fenstermacher JD (1983). Graphical evaluation of blood-to-brain transfer constants from multiple-time uptake data. J Cereb Blood Flow Metab.

[34] Stanisz GJ (2005). T1, T2 relaxation and magnetization transfer in tissue at 3T. Magn Reson Med.

[35] Pintaske J (2006). Relaxivity of Gadopentetate Dimeglumine (Magnevist), Gadobutrol (Gadovist), and Gadobenate Dimeglumine (MultiHance) in human blood plasma at 0.2, 1.5, and 3 tesla. Investig Radiol.

[36] Truijman MT (2013). Combined 18F-FDG PET-CT and DCE-MRI to assess inflammation and microvascularization in atherosclerotic plaques. Stroke.

[37] Kerwin WS (2008). Signal features of the atherosclerotic plaque at 3.0 tesla versus 1.5 tesla: impact on automatic classification. Journal of magnetic resonance imaging : JMRI.

[38] Derksen WJ (2011). Different stages of intraplaque hemorrhage are associated with different plaque phenotypes: a large histopathological study in 794 carotid and 276 femoral endarterectomy specimens. Atherosclerosis.

[39] McCarthy MJ (1999). Angiogenesis and the atherosclerotic carotid plaque: an association between symptomatology and plaque morphology. J Vasc Surg.

[40] Gossl M (2010). Segmental heterogeneity of vasa vasorum neovascularization in human coronary atherosclerosis. JACC Cardiovasc Imaging.

[41] Guo L (2018). CD163+ macrophages promote angiogenesis and vascular permeability accompanied by inflammation in atherosclerosis. J Clin Invest.

[42] Chen H (2013). Progression of experimental lesions of atherosclerosis: assessment by kinetic modeling of black-blood dynamic contrast-enhanced MRI. Magn Reson Med.

[43] Kerwin WS (2009). Contrast-enhanced MRI of carotid atherosclerosis: dependence on contrast agent. J Magn Reson Imaging.

[44] van Hoof RH (2017). Vessel wall and adventitial DCE-MRI parameters demonstrate similar correlations with carotid plaque microvasculature on histology. J Magn Reson Imaging.

[45] Falk E (1983). Plaque rupture with severe pre-existing stenosis precipitating coronary thrombosis. Characteristics of coronary atherosclerotic plaques underlying fatal occlusive thrombi. Br Heart J.

[46] Sun J (2016). Blood pressure is a major modifiable risk factor implicated in pathogenesis of Intraplaque hemorrhage: an in vivo magnetic resonance imaging study. Arterioscler Thromb Vasc Biol.

[47] Selwaness M (2013). Blood pressure parameters and carotid intraplaque hemorrhage as measured by magnetic resonance imaging: the Rotterdam study. Hypertension.

[48] Cicha I (2011). Carotid plaque vulnerability: a positive feedback between hemodynamic and biochemical mechanisms. Stroke.

[49] Huang X (2010). Intraplaque hemorrhage is associated with higher structural stresses in human atherosclerotic plaques: an in vivo MRI-based 3D fluid-structure interaction study. Biomed Eng Online.

[50] Lin R (2017). Association between carotid atherosclerotic plaque calcification and Intraplaque hemorrhage: a magnetic resonance imaging study. Arterioscler Thromb Vasc Biol.

[51] Zhongzhao T (2014). How does juxtaluminal calcium affect critical mechanical conditions in carotid atherosclerotic plaque? An exploratory study. IEEE Trans Biomed Eng.

[52] Kolodgie FD (2003). Intraplaque hemorrhage and progression of coronary atheroma. N Engl J Med.

[53] Underhill HR (2009). Arterial remodeling in [corrected] subclinical carotid artery disease. JACC Cardiovasc Imaging.

[54] Takaya N (2005). Presence of intraplaque hemorrhage stimulates progression of carotid atherosclerotic plaques: a high-resolution magnetic resonance imaging study. Circulation.

[55] Chen H (2013). Atherosclerotic plaque inflammation quantification using dynamic contrast-enhanced (DCE) MRI. Quant Imaging Med Surg.

[56] Parker GJ (2006). Experimentally-derived functional form for a population-averaged high-temporal-resolution arterial input function for dynamic contrast-enhanced MRI. Magn Reson Med.

[57] J Scott McNally S-EK, Mendes J, Rock Hadley J, Sakata A, De Havenon AH, Treiman GS, Parker DL (2017). Magnetic Resonance Imaging Detection of Intraplaque Hemorrhage. Magn Reson Insights.

[58] Moody AR (2003). Characterization of complicated carotid plaque with magnetic resonance direct thrombus imaging in patients with cerebral ischemia. Circulation.

[59] Kwee RM (2010). Carotid plaques in transient ischemic attack and stroke patients: one-year follow-up study by magnetic resonance imaging. Investig Radiol.

[60] Calcagno C (2013). The complementary roles of dynamic contrast-enhanced MRI and 18F-fluorodeoxyglucose PET/CT for imaging of carotid atherosclerosis. Eur J Nucl Med Mol Imaging.

[61] Wang J (2014). Varying correlation between 18F-Fluorodeoxyglucose positron emission tomography and dynamic contrast-enhanced MRI in carotid atherosclerosis: implications for plaque inflammation. Stroke.

[62] Sluimer JC (2008). Hypoxia, hypoxia-inducible transcription factor, and macrophages in human atherosclerotic plaques are correlated with intraplaque angiogenesis. J Am Coll Cardiol.

[63] Sluimer JC, Daemen MJ (2009). Novel concepts in atherogenesis: angiogenesis and hypoxia in atherosclerosis. J Pathol.

[64] Kwee RM (2012). Longitudinal MRI study on the natural history of carotid artery plaques in symptomatic patients. PLoS One.

